# The indirect effect of nonadherence on health-related quality of life in older adults with neurological disorders: implications for clinical endpoints and interventions

**DOI:** 10.3389/fneur.2024.1462478

**Published:** 2024-11-25

**Authors:** Aline Schönenberg, Sarah Mendorf, Tino Prell

**Affiliations:** ^1^Department of Geriatrics, Halle University Hospital, Halle, Germany; ^2^Department of Neurology, Jena University Hospital, Jena, Germany

**Keywords:** older adults, medication adherence, depression, health-related quality of life, quality of life

## Abstract

**Objective:**

This study assessed how Health-Related Quality of Life (HRQoL) and nonadherence to medication are linked, to determine whether HRQoL is a suitable endpoint for clinical trials evaluating nonadherence.

**Background:**

HRQoL is often used as an endpoint in clinical trials to determine the effectiveness of nonadherence interventions. However, the relationship between HRQoL and nonadherence is not clear, as some interventions find an effect of nonadherence on HRQoL while others do not. Since both HRQoL and nonadherence are latent constructs, it is of interest to understand the factors that link them.

**Methods:**

Medication nonadherence was assessed in 731 older adults with neurological disorders using the Stendal Adherence to Medication Score (SAMS). Regression and network analyses were performed to examine the association between the SAMS and HRQoL (SF-36). Cognitive function, depressive symptoms, mobility, and healthcare satisfaction were included as covariates.

**Results:**

There was a weak association between the SAMS and HRQoL only for the mental component scale. The relationship between the SAMS and HRQoL appears indirect, as its effect is nullified upon the inclusion of covariates, especially depressive symptoms. Network analyses showed that the effect of nonadherence on HRQoL is mainly delivered by depressive symptoms, while cognition and satisfaction with healthcare contribute to a lesser extent.

**Conclusion:**

Nonadherence and HRQoL are both latent variables influenced by similar factors. The effect of nonadherence on HRQoL seems to be indirect and mainly delivered by depressive symptoms, possibly via motivational pathways. These associations need to be considered when selecting clinical endpoints and planning interventions.

## 1 Introduction

Neurological disorders are the leading cause of disability worldwide, affecting more than 3 billion individuals (1). Most chronic diseases are treated with medications. In order for these medications to be effective, it is important that patients take them as prescribed (2, 3). However, a recent review (4) estimated that nearly 43% of patients show medication *nonadherence*, meaning that they do not take their medication as agreed with their healthcare providers (2, 5). Nonadherence to medication is associated with poorer health outcomes and subsequently reduced health-related quality of life (HRQoL) (6, 7). HRQoL depends, among other factors, on physical health status (8) but is also influenced by mental health, in particular depressive symptoms (9, 10). Depressive symptoms are also known to be predictors of nonadherence (11). In a systematic review, Yap et al. (12) cite both depressive symptoms and physical health, such as falls and poor physical function, as well as reduced cognitive function, as barriers to adherence.

HRQoL describes a person’s self-perceived health-related well-being and is therefore often used as an outcome in clinical trials to assess the effectiveness of health interventions. Cross et al. (13) provide a recent Cochrane review of nonadherence interventions in which 14 studies used an outcome measure of HRQoL with mixed results. For example, a pharmaceutical care intervention for older adults (14) failed to improve HRQoL. Similarly, an intervention study in patients with diabetes (15) found no significant effect on HRQoL after 6 months, neither did a study on patients with multimorbidity (16). While in the study by Willeboordse et al. (17), medication problems were solved for the intervention group, again no effect on HRQoL was found. In contrast, a nurse-led intervention produced an effect on both physical and mental components of HRQoL in geriatric patients (18). To put these findings into context, it is important to understand whether HRQoL is an appropriate outcome parameter for interventions targeting nonadherence in the first place. In cross-sectional studies, many studies report an effect of nonadherence on HRQoL (19–23) while others find no association (24, 25). Some studies also report that the effect of medication nonadherence is only evident for subscales (22) or disappears after controlling for certain covariates, especially depressive symptoms and mobility (21). This suggests that the effect of nonadherence on HRQoL is not straightforward and may depend on common influencing factors.

In a previous study, we found HRQoL to be an inappropriate variable for the identification of cut-off points for nonadherence (26). However, both in our data and in previous studies, nonadherence and HRQoL were influenced by similar factors, particularly depressive symptoms (9, 11, 12, 27). Depressive symptoms were also identified as the strongest predictor of nonadherence in the present dataset (28), therefore we hypothesize that depressive symptoms may connect nonadherence and HRQoL. As btoh HRQoL and nonadherence are important variables in clinical research, we aimed to understand how they are related. As nonadherence is often used as a modifiable means to influence HRQoL in interventions as well as an end-point to assess the effectiveness of these interventions, it is important to analyze how they are linked. Understanding whether nonadherence can directly improve HRQoL holds important implications for the planning and evaluation of clinical trials. In this analysis, we thus aim to understand whether nonadherence has a direct effect on HRQoL or whether other variables, particularly depressive symptoms, deliver this connection.

## 2 Materials and methods

### 2.1 Participants and recruitment

The data utilized in this analysis stems from the NeuroGerAdh study, which is described in detail in the respective data paper (28, 29). Briefly, older adults with neurological disorders were comprehensively assessed during their stay at the Department of Neurology at Jena University Hospital, Germany, between February 2019 and March 2020. Patients were included in the study if they were at least 60 years old (or 55 years old with multimorbidity), had a primary neurological diagnosis given by a neurology specialist, and gave written informed consent. The study was approved by the local ethics committee. Patients with severe depression (diagnosed Major Depressive Disorder), delirium or cognitive deficits were excluded (diagnosed dementia or Montreal Cognitive Assessment ≤18). For this analysis, all patients were included if they filled out the respective questionnaires of interest. The study utilized routine data in combination with questionnaires and assessments performed by trained study staff at baseline during the hospital stay, as well as telephone-based follow-up after 6 and 12 months to obtain information on survival and health. This analysis is based on the dataset collected at baseline including the following variables:

- Age (years), gender (male/female), marital status (married or in a relationship/single or widowed), living conditions (alone/not alone), education (grouped into low = ≤ 8 years, medium 9–11 years, high ≥12 years).
- Number of different drugs taken daily, main diagnosis grouped into 5 categories (Movement Disorder, Cerebrovascular Disorder, Neuromuscular Disorder, Epilepsy, Miscellaneous Disorders).
- Satisfaction with healthcare assessed with the Healthcare Climate Questionnaire (HCCQ) (30, 31).
- Personality assessed by the Big Five Inventory (BFI) (32).
- Cognition assessed with the Montreal Cognitive Assessment (MoCA) (33, 34).
- Mobility based on the Timed Up and Go (TuG) test (35).
- Depressive symptoms measured with Becks Depression Inventory II (BDI) (36, 37).

HRQoL was assessed using the 36-Item Short Form Health Survey (SF-36), with higher scores indicating higher HRQoL (38, 39). The SF-36 is the most commonly utilized valid patient-reported outcome measure for HRQoL (40). The eight subscales Vitality, Mental Health/ Emotional well-being (EWB), Social Functioning and Role Limitations due to Emotional Problems can be summarized into a Mental Component Scale (MCS), while the Physical Component Scale (PCS) encompasses the sub-scales General Health, Physical Functioning, Bodily Pain and Role Limitations due to Physical Problems (41, 42). To assess the impact of nonadherence, our analysis utilized MCS and PCS as dependent variables.

Nonadherence was measured using the Stendal Adherence to Medication Scale (SAMS), a 18-item self-report scale that encompasses the domains *Modification* of medication (for example *If you feel you have to take too many tablets, do you stop taking those medications you consider to be less important?*), *Forgetting* to take medication (such as *How often do you forget to take your medication?*), and missing *Knowledge* about medication (such as time of taking, dosage, or purpose). In this study, we analyzed the impact of the SAMS total score on HRQoL among the above mentioned covariates, and report subscale analyses in the Supplementary materials. Each item is posed as a 4-point Likert scale ranging from 0 to 4, with higher scores indicating higher levels of nonadherence. The SAMS was developed as an extension of the Morisky Medication Adherence Scale (MMAS) with more nuanced Likert scale items (88). It was constructed by an expert panel and incorporates items analogous to previously validated questionnaires, such as items about medication knowledge in accordance with Rottlaender et al. (90), as well as the Morisky scales (88, 89). Additional items were added by the expert panel of patients and healthcare providers. The final SAMS version has undergone testing across a range of patient groups, such as neurological patients, chronic pain patients, and patients who have received kidney transplants (43–49). Although self-reported measurements carry a risk of bias, they offer an opportunity to understand different types of nonadherence and their underlying causes, which cannot be achieved through the use of objective measures (50–53). Both objective and self-report measures of nonadherence have advantages and disadvantages (53, 54), and although objective measures are considered to be free of bias, patients must be informed that their medication intake will be monitored, potentially leading to increased adherence for the duration of the study. Likewise, prescription data or electronic pill counting cannot provide information on whether the medication was ingested, while analyses of drug concentration in the blood are difficult to implement outside of funded clinical research. As self-report measures show moderate to high correlation with objective measures, their use is recommended due to their economic application and informational value on different reasons for and types of nonadherence (3, 52, 54, 55).

### 2.2 Statistical analysis

As a first step, we report both mean and standard deviation (SD) as well as median and inter-quartile range (IQR), as Shapiro Wilk Test revealed non-normal distributions of the variables. Spearman correlations were calculated between the SAMS and the MCS/PCS scores as well as the covariates. Missing data in the covariates was treated with the pairwise deletion process. Of note, of the excluded patients, only 9 patients had more than 5 missing items in the SAMS whereas the majority missed out on one or two. Still, as the SAMS operates on an item level where each item contributes to the overall nonadherence score, we excluded all patients with missing data. All analyses were performed in R Version 4.3.0 (56) at a significance level of *p* = 0.05.

To assess whether the SAMS is related to the MCS and PCS, we performed linear regression first using only the SAMS as an independent variable, and secondly with the addition of the above-mentioned covariates. Assumptions for regression, such as collinearity, autocorrelation, homoscedasticity and normality of residuals, were assessed with the R-package *performance* (57).

In addition, we aimed to understand the interaction between SAMS, HRQoL and the covariates using Network Analysis (NA). NA is a tool that has recently been used extensively to study the bidirectional association between different variables (47, 58, 59). In our data, NA visualizes the partial correlations between variables while controlling for other associations in the network. Importantly, unlike traditional modeling, NA does not assume a singular direction of effect or an underlying latent variable. Instead, it suggests that the variables in the network influence each other in a circular manner (60–62). Networks contain two main components: the variables, called *nodes*, and their connections, called *edges* (63). Edges represent the strength of the relation between two nodes, with thicker edges visualizing stronger relationships and blue edges depicting positive associations, red negative ones. The nodes are positioned using the Fruchterman-Reingold algorithm based on the strengths of their associations (63). We performed NA with nonparametric bootstrap to assess network stability. The Correlation-stability coefficient (CSC) indicates the stability of a network if a portion of the participants is dropped and should remain above 0.5 (64). Although it is possible to calculate centrality indices using NA, we do not report these as the aim of our analysis was to show the flow of information between HRQoL, nonadherence and the covariates, not to assess which variable has the strongest influence in the network overall (65).

## 3 Results

### 3.1 Descriptive results

In total, *N* = 910 patients were recruited at baseline. Of those, *N* = 731 patients completed the measure of nonadherence and were thus included in the present analysis (Table 1). On average, they were 70.2 years old and 56.2% were female. They regularly took 5.74 different medications per day. The main neurological diagnoses were classified into movement, cerebrovascular, or neuromuscular disorders, epilepsy, and miscellaneous neurological diagnoses (see (29) for details) (Table 1).

**Table 1:** Description of the study participants.

Variable	M (SD)	MD (IQR)
Age	70.24 (8.61)	70 (14)
SAMS	6.16 (7.59)	4 (8)
BDI	9.68 (7.51)	8 (9)
MoCA	23.51 (2.71)	23 (4)
TuG	10.65 (4.48)	10 (4)
HCCQ	5.62 (1.13)	5.93 (1.27)
Number of drugs	5.74 (3.68)	5 (5)
MCS	48.95 (11.04)	50.74 (18.07)
PCS	33.87 (11.09)	33.09 (16.47)
	Count	%
Gender		
Male	405	56.17
Female	326	42.21
Education		
Low	224	31.37
Medium	249	34.87
High	251	35.15
Living situation		
Alone	172	24.61
Not alone	527	75.39
Diagnosis		
Movement disorder	237	32.87
Cardiovascular disorder	191	26.49
Neuromuscular disorder	143	19.83
Epilepsy	35	4.85
Miscellaneous	125	17.34
BFI		
Neuroticism	81	11.56
Openness	114	16.26
Agreeableness	60	8.56
Conscientiousness	298	42.51
Extraversion	148	21.11

M, Mean; SD, Standard Deviation; MD, Median; IQR, Interquartile Range. BDI, Beck Depression Inventory; BFI, Big Five Inventory; SAMS, Stendal Adherence to Medication Scale; MoCA, Montreal Cognitive Assessment; TuG, Time up and Go Test; HCCQ, Healthcare Climate Questionnaire; MCS, SF-36 Mental Component Scale; PCS, SF-36 Physical Component Scale.

### 3.2 Nonadherence and HRQoL

We then analyzed how the SAMS was related to HRQoL as assessed by the MCS and the PCS of the SF-36, which exhibited a Cronbach’s alpha of 0.89 each in our dataset. Spearman correlations (Supplementary Table 1) showed that the SAMS was weakly correlated with the MCS (*r* = −0.20, *p* < 0.001) and the PCS (*r* = −0.13, *p* < 0.001), as well as with other SF-36 subscales (r ranging from −0.13 for the physical subscale to −0.19 for the emotional and social subscales). These results already suggest a negative association between nonadherence and HRQoL, with a stronger association with the mental scales than with the physical scales. In addition, the SAMS was most strongly correlated with the BDI (*r* = 0.30, *p* < 0.001), as well as the TuG (*r* = 0.12, *p* = 0.009) and negatively with the MoCA (*r* = −0.13, *p* = 001) and the HCCQ (*r* = −0.19, *p* < 0.001). This indicates that higher levels of depressive symptoms, worse mobility, worse cognition and worse healthcare satisfaction are associated with higher levels of nonadherence.

We confirmed the relationship between the SAMS and the MCS using a simple linear regression model (Table 2). The SAMS was found to be a significant contributor to the MCS (est. = −0.028, CI [−0.038, −0.017], *p* < 0.001), explaining 3.3% of the MCS variance. However, this association between the SAMS and the MCS disappeared when covariates were added to the model; instead, the BDI, number of drugs, HCCQ, and MoCA were identified as the factors explaining most variance in MCS (Table 2).

**Table 2:** Linear regression for the MCS with (a) SAMS and (b) SAMS and covariates.

A	MCS		
Predictors	Estimates	CI	*p*
(Intercept)	50.62	49.58–51.67	<0.001
SAMS	−0.28	−0.38 – −0.17	<0.001
*N* = 689, *R*^2^ / *R*^2^ adjusted = 0.035 / 0.033			
**B**	**MCS**		
(Intercept)	26.52	13.72–39.32	**<0.001**
SAMS	0.09	−0.04 – 0.22	0.160
BDI	−0.92	−1.05 – −0.79	**<0.001**
TuG	0.18	−0.00 – 0.37	0.056
Number of drugs per day	0.44	0.21–0.67	**<0.001**
Age	0.00	−0.10 – 0.10	0.974
MoCA	0.60	0.28–0.92	**<0.001**
HCCQ	1.07	0.33–1.81	**0.005**
Gender: female	0.35	−1.31 – 2.00	0.680
Cardiovascular disorder	1.81	−0.46 – 4.08	0.118
Neuromuscular disorder	2.07	−0.12 – 4.25	0.064
Epilepsy	1.60	−2.31 – 5.52	0.422
Miscellaneous disorder	2.58	0.27–4.89	**0.029**
Living with partner	0.43	−1.49 – 2.35	0.659
Education medium	1.93	−0.11 – 3.98	0.064
Education high	−0.75	−2.78 – 1.29	0.471
BFI: Openness	1.85	−1.28 – 4.99	0.246
BFI: Agreeableness	2.16	−1.51 – 5.83	0.248
BFI: Conscientiousness	4.61	1.91–7.31	**0.001**
BFI: Extraversion	4.06	0.99–7.13	**0.010**

*N = 397; R2 / R2 adjusted = 0.499 / 0.473. VIF ≤ 1.41 for all variables.*

CI, 95% confidence interval; SAMS, Stendal Adherence to Medication Scale; BDI, Beck Depression Inventory; TuG, Time up and Go Test; MoCA, Montreal Cognitive Assessment; HCCQ, Healthcare Climate Questionnaire; BFI, Big Five Inventory – in reference to Neuroticism; MCS, Mental Component Scale of SF-36; Diagnosis – in reference to Movement Disorder.

Statistically significant values *p* < 0.05 are presented in bold.

For the PCS, the SAMS was not significantly associated even in the simple model (*p* = 0.104), see Supplementary Table 2.1. Detailed analyses for the SAMS and other subscales of the SF-36 are given in Supplementary Tables 2.2–2.9, showing that the SAMS was more strongly associated with the mental subscales. It is worth noting that in all models, the initial influence of the SAMS diminishes when covariates are added into the model. Since the PCS is not significantly associated with the SAMS, we focused on the MCS alone, as the mental subscales are more closely associated with nonadherence in our data.

**Figure 1:**
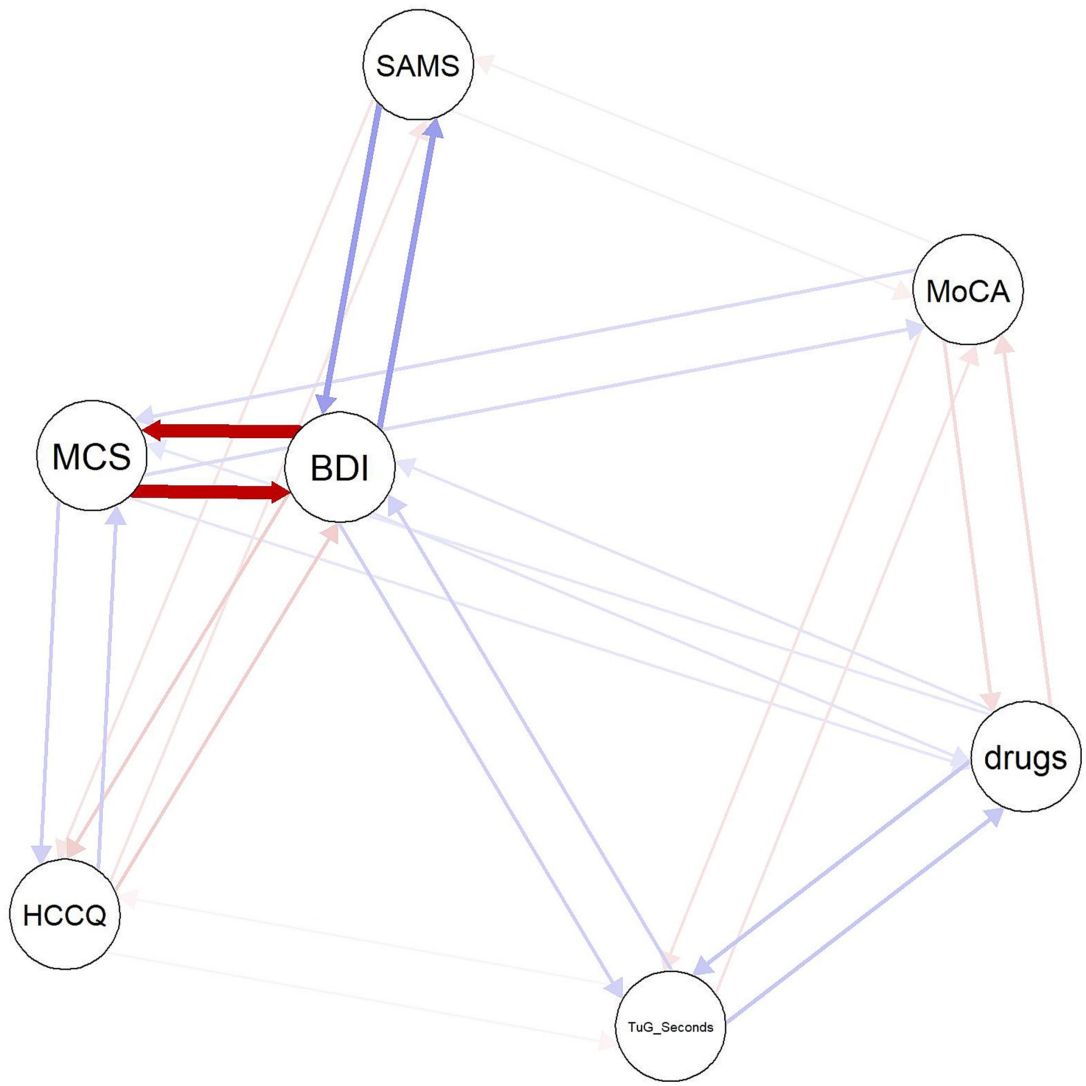
Network plot of SAMS, MCS and covariates. BDI, Beck Depression Inventory; drugs, number of drugs; HCCQ, Healthcare Climate Questionnaire; MCS, SF-36 Mental Component Score; MoCA, Montreal Cognitive Assessment; SAMS, Stendal Adherence to Medication Score; TuG_Seconds, Timed Up and Go. Red lines depict negative, blue lines positive associations.

In our previous analysis of this dataset, we found a strong association between the SAMS and the BDI, but also between the SAMS and mobility, cognition, number of medications, age, and HCCQ (28). Our present analyses confirm that when these variables are included, the association between the SAMS and the MCS disappears. Thus, it is likely that these variables act as a link between MCS and SAMS. Therefore, we performed a network analysis to obtain an overview of the relationships between the SAMS and the variables identified as significant in the regression models (Figure 1). The network is stable with a CSC = 0.75 and presents 7 nodes connected by 28 out of 49 possible edges. It shows bidirectional relationships between SAMS and BDI, HCCQ, and MoCA. As expected, there is no direct connection between SAMS and MCS. Instead, visual inspection shows that the SAMS is connected to the MCS via the BDI, which has the strongest connection to the MCS, as well as via HCCQ and MoCA. The relationship with TuG and Number of Drugs is not straightforward in the network, instead they are connected through BDI and MoCA.

Finally, to confirm the association between the MCS and the variables included in the NA, we lastly performed linear regressions including the SAMS as well as each individual variable from the network. Notably, the SAMS remained significant as an independent variable to explain variance in the MCS when only HCCQ, TuG, number of drugs, and MoCA were included (Supplementary Table 3). However, once the BDI was included (Table 3), the significant influence of the SAMS disappeared (est = −0.04, *p* = 0.349, CI [−0.13, 0.05]).

**Table 3:** Linear regression on the mental component scale using the SAMS and BDI as dependent variables.

A	MCS		
Predictors	Estimates	CI	*p*
(Intercept)	57.87	56.76–58.98	**<0.001**
SAMS	−0.04	−0.13 – 0.05	0.349
BDI	−0.90	−0.99 – −0.81	**<0.001**

*N = 689; R2 adjusted = 0.381.*

BDI, Beck Depression Inventory; MCS, Mental Component Scale of SF-36; SAMS, Stendal Adherence to Medication Score; CI, 95% confidence interval.

Statistically significant values *p* < 0.05 are presented in bold.

As the SAMS can further be classified into nonadherence subscales, we repeated the analyses using the subscales *Forgetting* to take medication, *Modification* of medication intake, and missing *Knowledge* about medication (Supplementary Tables 4–6). Linear regression revealed that the effect of *Forgetting* on the MCS disappears (*p* = 0.068) after adding the BDI (*p* < 0.001) or HCCQ (*p* < 0.001) to the model. Likewise, including the BDI (*p* < 0.001) nullifies the effect of missing *Knowledge* (*p* = 0.368) and *Modification* (*p* = 0.103) on the MCS, whereas the inclusion of HCCQ, Number of Medications, TuG or MoCA do not annul the association between SAMS and MCS.

## 4 Discussion

The aim of our analysis was to evaluate the effect of nonadherence on HRQoL and to determine the suitability of HRQoL as an outcome for clinical trials targeting nonadherence. Using regression and network analysis, we found a weak association between the mental subscales of HRQoL (SF-36) and nonadherence, which was mainly delivered via depressive symptoms.

Of note, a weak association between HRQoL and nonadherence has been reported previously (18, 25), while other studies have found stronger associations (20–22). Differences in the covariates and patients included in the studies may explain these variations. In addition, nonadherence represents a highly complex, multi-faceted phenomenon (66), and interventions that only address specific aspects of nonadherence may prove insufficient to influence HRQoL (13). Our research further suggests that other factors, particularly depressive symptomology, may connect nonadherence and HRQoL, making it an indirect link. Although medication nonadherence may impact health especially in older, chronically ill patients that depend on their pharmacotherapy, (HR)QoL encompasses more than mere physical health. Especially in advancing age, where a full recovery is not feasible and a certain health decline can be expected, other factors such as satisfaction with life, social connection and purpose take precedence (67, 68). Likewise, HRQoL describes a person’s satisfaction with and interpretation of their health status, thus it may be independent of the objective health. The paradox that some persons report high HRQoL despite being physically ill may be explained by differences in expectations regarding health and aging that strongly influence a person’s interpretation of their health status (69, 70). Importantly, the gap between desired and present health status is also influenced by depressive symptoms (69).

Overall, the relationship between depressive symptoms and nonadherence is well documented in the literature (11, 12, 27, 28). Depressive symptoms are associated with reduced self-efficacy, loss of control and interest as well as hopelessness and fatigue (3, 27, 71, 72). This may lead to changes in the beliefs about the effectiveness of medication: if someone does not believe that they can make a positive difference to their health, they may feel that taking their medication is less necessary (73). Thus, depressive symptoms may affect patients’ motivation to take medication (74). This strong association between nonadherence and depressive symptoms is further highlighted by our subscale analyses, showing that depressive symptoms not only influence overall nonadherence but also the three subscales *Forgetting* to take medication, missing *Knowledge* about medication, and *Modification* of medication. In a previous publication based on this dataset, we performed a symptom-driven analysis to understand which depressive symptoms in particular drive the association with nonadherence. Loss of interest, fatigue and difficulties with concentration tie depressive symptoms to nonadherence for all subscales, indicating an overall lack of investment in one’s own health that may lead to carelessness with medication intake (27). In addition, Lee and Oh (8) showed that self-efficacy and emotional support reduce nonadherence, both of which may be reduced in patients with higher levels of depressive symptoms (75, 76). Schoenthaler et al. (76) conducted a mediation analysis showing that self-efficacy mediates the relationship between depressive symptoms and nonadherence. Similarly, Chantzaras and Yfantopoulos (21) found that the relationship between HRQoL and nonadherence was determined by the level of self-care, again strengthening the relation between nonadherence and depressive symptoms via motivational and behavioral paths. Likewise, depressive symptoms are associated with psychosocial parameters such as loneliness and reduced social participation, as well as with worse physical health, all of which may influence HRQoL (9, 77, 78). Thus, depressive symptoms have the potential to simultaneously impact nonadherence and HRQoL. Future studies may benefit from longitudinal data on nonadherence, HRQoL and depressive symptoms to conduct mediation analyses and allow for causal interpretation to confirm this hypothesis.

Additionally, poor healthcare climate and cognitive deficits have previously been identified as barriers to adherence. Our NA supports this link, demonstrating connecting effects through HCCQ and MoCA in the network (12, 13). Healthcare climate is associated with patient engagement and better communication with healthcare providers, leading to better health-literacy and understanding of prescribed medications (12, 13). On the other hand, cognitive deficits may pose a challenge to understanding medication regimens and may lead to forgetfulness (12, 13). However, as shown in our regression and network analysis, the effects of cognition and healthcare climate are comparatively smaller than those of depressive symptoms and cannot fully explain the association between nonadherence and HRQoL. Of note, we excluded patients with severe dementia from study participation, thus this association may be stronger in a patient population with dementia (79).

Our results further suggest that the relationship between nonadherence, HRQoL and mobility (TuG) or number of drugs is not straightforward. While both mobility and number of drugs as a proxy for health (in terms of multimorbidity) are associated with HRQoL (8), they did not nullify the association between HRQoL and nonadherence in our data the way the BDI did. On average, our patients were taking 5.74 medications daily. Although increased treatment complexity correlates with nonadherence, the majority of our patients had chronic conditions, suggesting that the habitual use of their medication could mitigate the effect of complexity on nonadherence (8, 12, 80, 81). Likewise, different types of nonadherence are differentially associated with treatment complexity, as a higher number of medications may increase unintentional forgetting of medication but not intentional modification of their intake (28). In addition, although mobility limitations can affect HRQoL, they were not severe enough to interfere with medication use in our patients (21).

Overall, we found a stronger effect of nonadherence on the mental rather than on physical aspects of HRQoL. This has been confirmed in previous research (12, 18, 21). For example, in their review, Hickey et al. report a decreasing association between physical health and HRQoL with advandcing age (82). Likewise, Bernsten et al. (14) attribute the effect of mental health to psychosocial pathways such as increased support. One explanation suggested by our data is the close connection between nonadherence and motivation, self-efficacy and health beliefs through depressive symptoms, which are captured in the mental subscales of the SF-36 (27). These results are also consistent with the stronger association of nonadherence with depressive symptoms than with physical health such as mobility and multimorbidity found in our analysis. This again points to the strong influence of depressive symptoms on both nonadherence and HRQoL (9, 12).

Overall, meta-analyses show that previous intervention studies aimed at improving nonadherence have often been ineffective, which may be attributed in part to methodological variations in the included studies (13). Similarly, both HRQoL and nonadherence are complex behaviors with multiple components that need to be aligned for an effective intervention, as shown by the finding that multifaceted interventions yield the best results (13, 83). However, another reason as to why interventions remain underperforming may be that in order to assess the effectiveness of a clinical trial, it is essential to identify an appropriate endpoint. In their review, Cross et al. (13) provided an overview of the quality of evidence depending on the chosen outcome. Previous studies have found that interventions are more effective when changes in nonadherence itself, rather than HRQoL, are considered as an endpoint (83). Our analyses combined with previous research suggest that the failure to find an effect on HRQoL does not imply that the intervention itself was ineffective. Rather, it is plausible that nonadherence alone cannot sufficiently influence HRQoL because they are indirectly linked. Instead, it is critical to use outcomes that are directly related to nonadherence to measure the effectiveness of interventions targeting medication adherence. Additionally, in the design and evaluation of clinical trials on medication adherence and HRQoL, particular attention should be paid to depressive symptoms, especially to the connecting symptoms such as lack of motivation, interest in one’s health, fatigue and cognitive overload. As they appear to be linking nonadherence with HRQoL, overcoming barriers in motivational and behavioral pathways related to depressive symptoms can enable patients to take an active role in their healthcare (84, 85).

### 4.1 Limitations

As we were interested in the type of nonadherence we used a self-report measure, although it is susceptible to bias. However, objective measures of nonadherence have other shortcomings (see (3) for a discussion), and research has shown that using a validated scale can provide data comparable to objective measures (86). Still, the nonadherence data used in this study was not validated against an objective measure, thus it should be interpreted with caution. As there is no generally accepted cut-off point for nonadherence, we treated the SAMS as a continuous variable (3).

In addition, the single-center and cross-sectional design of our analysis reduces the generalizability and causal interpretation of our results, as does the inclusion of a specific patient population. Results may be different for other patients in different countries. Additionally, although many relevant covariates were included in our study, both HRQoL and nonadherence are complex constructs influenced by a multitude of factors, future studies should assess both using different scales. Especially in research on older adults, age-related changes should be appropriately considered in the HRQoL- instrument (82). Additional factors such as anxiety, social support and measures of health or daily activities should be included in future studies. Likewise, treatment burden and adverse health events impact on satisfaction with healthcare and adherence, as they may influence the perceived effectiveness of the treatment (87). These variables should be incorporated in future studies to understand which other factors connect nonadherence and HRQoL.

## 5 Conclusion

Our findings, along with past research, indicate that HRQoL is not suitable as a single endpoint for clinical trials to improve nonadherence, as there is no straightforward effect of nonadherence on HRQoL in this patient population. However, ours and previous analyses revealed that nonadherence and HRQoL are connected by similar variables, predominantly depressive symptomology. Depressive symptoms seem to provide a link between nonadherence and HRQoL, potentially via motivational pathways. When designing interventions on HRQoL and medication-taking behavior, it is essential to pay close attention to depressive symptomatology and further related covariates.

## Data Availability

The dataset presented in this study can be found in online repositories, an anonymous version is freely available for noncommercial scientific purposes from Prell et al. (29).
