# Supplementary material for: The indirect effect of nonadherence on health-related quality of life in older adults with neurological disorders: implications for clinical endpoints and interventions
Source: Front Neurol. 2024 Nov 25;15:1462478. doi: 10.3389/fneur.2024.1462478 (PMC11626440; doi:10.3389/fneur.2024.1462478)
Supplement: Supplementary file 1 [file Supplementary_file_1.pdf]

Supplement to: The indirect effect of Nonadherence on Health-Related Quality of Life in Older Adults with Neurological Disorders: Implications for Clinical Endpoints and Interventions

Supplementary Material

Supplement Table 1. **Spearman correlation of SAMS with other variables in the dataset**

	**MCS**	**PCS**	**Vitality**	**Health**	**Phys**	**Emot**	**EWB**	**Soc**	**TuG**	**Drugs**
**r**	-.20	-.13	-.18	-.18	-.13	-.19	-.18	-.19	.12	.19
p	< .001	< .001	< .001	< .001	< .001	< .001	< .001	< .001	.009	< .001
	**BDI**	**Age**	**MoCA**	**HCCQ**	**Educ.**	**Diag.**	**BFI**	**Marital**	**Living**	**Sex**
**r**	.30	.03	-.13	-.19	-.01	-.10	.07	.06	.02	.13
p	< .001	.369	.001	< .001	.760	.008	.049	.123	.565	< .001

Note: MCS = SF-36 Mental Component Score, PCS = SF-36 Physical Component Score, Vitality = SF-36 Vitality Subscale, Health = SF-36 General Health Subscale, Phys = SF-36 Physical Functioning, Emot = SF-36 Role Limitations Emotional Subscale, EWB = SF-36 Emotionall Well-Being, Soc = SF-36 Social Functioning, TuG = Timed Up And Go (Mobility), Drugs = Number of different Medications taken regularly, BDI = Becks Depression Inventory, HCCQ = Healthcare Climate Questionnaire, MoCA = Montreal Cognitive Assessment, Educ = Education, BFI = Big Five Inventory, Marit = Marital Status, Living = Living Situation

Supplement Tables 2.1-2.9. Linear Regression of SAMS on other SF-36 domains

1 Physical Component Scale

	PCS		
Predictors	*Estimates*	*CI*	*p*
(Intercept)	34.43	33.36 – 35.50	**<0.001**
SAMS	-0.09	-0.20 – 0.02	0.104

N = 689, R^2 / R^2 adjusted = 0.004 / 0.002

PCS = SF-36 Physical Component Scale, SAMS = Stendal Adherence To Medication Score

2 Vitality

	SF-36 Vitality		
Predictors	*Estimates*	*CI*	*p*
(Intercept)	50.03	48.17 – 51.89	**<0.001**
SAMS	-0.29	-0.48 – -0.09	**0.004**

N = 725, R^2 / R^2 adjusted = 0.012 / 0.010

	SF-36 Vitality		
Predictors	*Estimates*	*CI*	*p*
(Intercept)	52.84	27.35 – 78.34	**<0.001**
SAMS	0.23	-0.02 – 0.48	0.073
BDI	-1.44	-1.70 – -1.18	**<0.001**
TuG Seconds	-0.53	-0.90 – -0.16	**0.005**

Supplement to: The indirect effect of Nonadherence on Health-Related Quality of Life in Older Adults with Neurological Disorders: Implications for Clinical Endpoints and Interventions

Number of Drugs	-0.52	-0.97 – -0.07	**0.024**
Age	0.06	-0.14 – 0.27	0.529
MoCA	0.35	-0.28 – 0.97	0.279
HCCQ	-0.16	-1.62 – 1.30	0.827
Gender: Female	2.06	-1.24 – 5.36	0.220
Cardiovascular disorder	4.15	-0.36 – 8.66	0.071
Neuromuscular disorder	-1.14	-5.51 – 3.23	0.608
Epilepsy	5.94	-1.88 – 13.76	0.136
Miscellaneous disorder	-0.96	-5.54 – 3.63	0.683
Living with Partner	-0.34	-4.12 – 3.44	0.859
Education Medium	2.91	-1.17 – 6.99	0.161
Education High	-2.20	-6.25 – 1.85	0.287
BFI: Openness	3.59	-2.67 – 9.85	0.260
BFI: Agreeableness	-3.06	-10.39 – 4.28	0.413
BFI: Conscientiousness	5.12	-0.29 – 10.53	0.063
BFI: Extraversion	6.40	0.28 – 12.53	**0.041**

N = 421, R^2 / R^2 adjusted = 0.370 / 0.340

Note: SAMS = Stendal Adherence to Medication Scale, BDI = Beck Depression Inventory, TuG = Time up and Go Test, MoCA = Montreal Cognitive Assessment, HCCQ = Healthcare Climate, Questionnaire, BFI = Big Five Inventory

3 General Health

	**SF-36 General Health**		
Predictors	*Estimates*	*CI*	*p*
(Intercept)	45.89	44.33 – 47.45	**<0.001**
SAMS	-0.25	-0.42 – -0.09	**0.002**
Observations	722		
R^2 / R^2 adjusted	0.013 / 0.012		

	**SF-36 General Health**		
Predictors	*Estimates*	*CI*	*p*
(Intercept)	55.44	33.44 – 77.43	**<0.001**
SAMS	0.09	-0.12 – 0.31	0.402
BDI	-0.94	-1.16 – -0.72	**<0.001**
TuG Seconds	-0.44	-0.76 – -0.12	**0.006**
Number of Drugs	-0.65	-1.04 – -0.26	**0.001**
Age	0.10	-0.07 – 0.28	0.255
MoCA	0.01	-0.53 – 0.55	0.966
HCCQ	0.02	-1.24 – 1.27	0.979
Gender: Female	-4.00	-6.85 – -1.16	**0.006**
Cardiovascular disorder	2.08	-1.83 – 6.00	0.296
Neuromuscular disorder	-3.79	-7.56 – -0.02	**0.049**
Epilepsy	4.12	-2.60 – 10.84	0.229
Miscellaneous disorder	-1.49	-5.44 – 2.47	0.460
Living with Partner	-1.24	-4.51 – 2.04	0.458

Education Medium	2.98	-0.53 – 6.50	0.096
Education High	-0.29	-3.79 – 3.21	0.872
BFI: Openness	2.56	-2.82 – 7.94	0.350
BFI: Agreeableness	-0.55	-6.85 – 5.75	0.865
BFI: Conscientiousness	2.12	-2.54 – 6.77	0.372
BFI: Extraversion	4.65	-0.62 – 9.92	0.083

N = 417, R^2 / R^2 adjusted = 0.289 / 0.255

Note: SAMS = Stendal Adherence to Medication Scale, BDI = Beck Depression Inventory, TuG = Time up and Go Test, MoCA = Montreal Cognitive Assessment, HCCQ = Healthcare Climate, Questionnaire, BFI = Big Five Inventory

4 Physical Functioning

	**SF-36 Physical Functioning**		
Predictors	*Estimates*	*CI*	*p*
(Intercept)	50.10	47.19 – 53.00	**<0.001**
SAMS	-0.34	-0.63 – -0.04	**0.026**

N = 728, R^2 / R^2 adjusted = 0.007 / 0.005

	**SF-36 Physical Functioning**		
Predictors	*Estimates*	*CI*	*p*
(Intercept)	98.03	60.92 – 135.13	**<0.001**
SAMS	-0.06	-0.42 – 0.30	0.731
BDI	-1.32	-1.69 – -0.96	**<0.001**
TuG Seconds	-2.20	-2.73 – -1.67	**<0.001**
Number of Drugs	-1.88	-2.54 – -1.23	**<0.001**

Age	0.14	-0.15 – 0.43	0.352
MoCA	0.25	-0.66 – 1.17	0.586
HCCQ	-2.05	-4.19 – 0.08	0.059
Gender: Female	5.31	0.54 – 10.08	**0.029**
Cardiovascular disorder	4.67	-1.86 – 11.21	0.161
Neuromuscular disorder	-9.49	-15.82 – -3.15	**0.003**
Epilepsy	6.45	-4.85 – 17.76	0.262
Miscellaneous disorder	-3.53	-10.17 – 3.12	0.297
Living with Partner	-7.39	-12.87 – -1.92	**0.008**
Education Medium	3.26	-2.65 – 9.18	0.279
Education High	0.90	-4.95 – 6.75	0.763
BFI: Openness	-1.55	-10.56 – 7.46	0.735
BFI: Agreeableness	-6.00	-16.68 – 4.68	0.270
BFI: Conscientiousness	2.49	-5.33 – 10.30	0.532
BFI: Extraversion	-1.98	-10.86 – 6.90	0.661

N = 420, R^2 / R^2 adjusted = 0.400 / 0.372

Note: SAMS = Stendal Adherence to Medication Scale, BDI = Beck Depression Inventory, TuG = Time up and Go Test, MoCA = Montreal Cognitive Assessment, HCCQ = Healthcare Climate, Questionnaire, BFI = Big Five Inventory

5 Role Limitations due to Emotional Functioning

	**SF-36 Role Limitations Emotional Functioning**		
Predictors	*Estimates*	*CI*	*p*
(Intercept)	69.89	65.67 – 74.11	**<0.001**

SAMS	-1.08	-1.51 – -0.65	**<0.001**

N = 706, R^2 / R^2 adjusted = 0.033 / 0.032

SF-36 Role Limitations Emotional Functioning

Predictors	*Estimates*	*CI*	*p*
(Intercept)	-0.47	-63.24 – 62.31	0.988
SAMS	-0.03	-0.65 – 0.59	0.929
BDI	-2.80	-3.45 – -2.16	**<0.001**
TuG Seconds	0.07	-0.86 – 1.00	0.881
Number of Drugs	0.50	-0.61 – 1.62	0.373
Age	-0.09	-0.59 – 0.41	0.722
MoCA	2.61	1.05 – 4.16	**0.001**
HCCQ	3.82	0.23 – 7.42	**0.037**
Gender: Female	-2.58	-10.75 – 5.60	0.536
Cardiovascular disorder	12.35	1.23 – 23.46	**0.030**
Neuromuscular disorder	1.88	-8.84 – 12.60	0.730
Epilepsy	6.85	-12.56 – 26.26	0.488
Miscellaneous disorder	11.88	0.54 – 23.21	**0.040**
Living with Partner	-1.41	-10.80 – 7.98	0.768
Education Medium	10.08	0.01 – 20.16	**0.050**
Education High	8.73	-1.23 – 18.70	0.086
BFI: Openness	-1.94	-17.39 – 13.52	0.805
BFI: Agreeableness	-3.21	-21.30 – 14.89	0.728

BFI: Conscientiousness	1.08	-12.31 – 14.48	0.874
BFI: Extraversion	0.51	-14.66 – 15.68	0.948

N = 407, R^2 / R^2 adjusted = 0.287 / 0.252

Note: SAMS = Stendal Adherence to Medication Scale, BDI = Beck Depression Inventory, TuG = Time up and Go Test, MoCA = Montreal Cognitive Assessment, HCCQ = Healthcare Climate, Questionnaire, BFI = Big Five Inventory

6 Emotional Well-Being

	**SF-36 Emotional Wellbeing**		
Predictors	*Estimates*	*CI*	*p*
(Intercept)	67.86	66.08 – 69.63	**<0.001**
SAMS	-0.42	-0.61 – -0.24	**<0.001**

N = 725, R^2 / R^2 adjusted = 0.027 / 0.026

	**SF-36 Emotional Wellbeing**		
Predictors	*Estimates*	*CI*	*p*
(Intercept)	53.60	31.98 – 75.23	**<0.001**
SAMS	0.18	-0.03 – 0.39	0.096
BDI	-1.59	-1.81 – -1.38	**<0.001**
TuG Seconds	-0.30	-0.62 – 0.01	0.058
Number of Drugs	0.49	0.10 – 0.87	**0.013**
Age	-0.02	-0.19 – 0.15	0.846
MoCA	0.56	0.03 – 1.10	**0.038**
HCCQ	1.01	-0.22 – 2.25	0.108
Gender: Female	2.34	-0.46 – 5.14	0.101

Cardiovascular disorder	2.24	-1.59 – 6.06	0.251
Neuromuscular disorder	0.90	-2.81 – 4.61	0.633
Epilepsy	3.80	-2.84 – 10.43	0.261
Miscellaneous disorder	2.97	-0.93 – 6.86	0.135
Living with Partner	-0.87	-4.08 – 2.34	0.593
Education Medium	1.53	-1.93 – 4.99	0.385
Education High	0.08	-3.36 – 3.52	0.963
BFI: Openness	3.91	-1.40 – 9.22	0.149
BFI: Agreeableness	2.80	-3.42 – 9.02	0.377
BFI: Conscientiousness	9.30	4.71 – 13.89	**<0.001**
BFI: Extraversion	6.10	0.91 – 11.30	**0.021**

N = 421, R^2 / R^2 adjusted = 0.490 / 0.465

Note: SAMS = Stendal Adherence to Medication Scale, BDI = Beck Depression Inventory, TuG = Time up and Go Test, MoCA = Montreal Cognitive Assessment, HCCQ = Healthcare Climate, Questionnaire, BFI = Big Five Inventory

7 Social Functioning

	SF-36 Social Functioning		
Predictors	*Estimates*	*CI*	*p*
(Intercept)	76.21	73.72 – 78.70	**<0.001**
SAMS	-0.67	-0.92 – -0.41	**<0.001**

N = 730, R^2 / R^2 adjusted = 0.035 / 0.034

SF-36 Social Functioning

Predictors	*Estimates*	*CI*	*p*
(Intercept)	41.03	8.86 – 73.20	**0.013**
SAMS	-0.14	-0.45 – 0.18	0.393
BDI	-1.85	-2.17 – -1.53	**<0.001**
TuG Seconds	-0.17	-0.64 – 0.29	0.462
Number of Drugs	-0.14	-0.71 – 0.43	0.628
Age	0.29	0.03 – 0.54	**0.028**
MoCA	0.81	0.02 – 1.60	**0.045**
HCCQ	0.79	-1.05 – 2.63	0.399
Gender: Female	3.64	-0.53 – 7.80	0.087
Cardiovascular disorder	4.94	-0.76 – 10.63	0.089
Neuromuscular disorder	-1.62	-7.13 – 3.90	0.564
Epilepsy	7.53	-2.33 – 17.39	0.134
Miscellaneous disorder	-0.10	-5.89 – 5.69	0.973
Living with Partner	0.33	-4.44 – 5.10	0.892
Education Medium	3.29	-1.86 – 8.44	0.209
Education High	-8.57	-13.68 – -3.47	**0.001**
BFI: Openness	5.24	-2.63 – 13.10	0.191
BFI: Agreeableness	2.95	-6.30 – 12.20	0.531
BFI: Conscientiousness	9.80	2.98 – 16.62	**0.005**
BFI: Extraversion	8.95	1.22 – 16.67	**0.023**

N = 422, R^2 / R^2 adjusted = 0.418 / 0.391

Note: SAMS = Stendal Adherence to Medication Scale, BDI = Beck Depression Inventory,

TuG = Time up and Go Test, MoCA = Montreal Cognitive Assessment, HCCQ = Healthcare Climate, Questionnaire, BFI = Big Five Inventory

8 Pain

SF-36 Pain

Predictors	*Estimates*	*CI*	*p*
(Intercept)	55.94	53.04 – 58.85	**<0.001**
SAMS	-0.22	-0.51 – 0.08	0.154

N = 730, R^2 / R^2 adjusted = 0.003 / 0.001

9 Role Limitations due to Physical Functioning

SF-36 Role Limitations Physical Functioning

Predictors	*Estimates*	*CI*	*p*
(Intercept)	35.09	31.34 – 38.85	**<0.001**
SAMS	-0.83	-1.22 – -0.45	**<0.001**

N = 705, R^2 / R^2 adjusted = 0.025 / 0.023

SF-36 Role Limitations Physical Functioning

Predictors	*Estimates*	*CI*	*p*
(Intercept)	24.44	-33.38 – 82.26	0.406
SAMS	-0.20	-0.78 – 0.38	0.496
BDI	-2.01	-2.61 – -1.42	**<0.001**
TuG Seconds	-1.13	-1.98 – -0.27	**0.010**
Number of Drugs	-1.69	-2.72 – -0.67	**0.001**

Age	0.37	-0.09 – 0.83	0.112
MoCA	0.92	-0.51 – 2.36	0.207
HCCQ	-0.51	-3.83 – 2.80	0.761
Gender: Female	2.36	-5.17 – 9.90	0.538
Cardiovascular disorder	13.87	3.65 – 24.09	**0.008**
Neuromuscular disorder	-3.69	-13.62 – 6.23	0.465
Epilepsy	30.66	12.77 – 48.55	**0.001**
Miscellaneous disorder	9.01	-1.51 – 19.53	0.093
Living with Partner	8.31	-0.37 – 16.99	0.061
Education Medium	3.97	-5.30 – 13.24	0.400
Education High	-0.84	-10.06 – 8.38	0.859
BFI: Openness	-11.81	-26.09 – 2.46	0.105
BFI: Agreeableness	-29.61	-46.28 – -12.94	**0.001**
BFI: Conscientiousness	-9.43	-21.76 – 2.90	0.133
BFI: Extraversion	-10.35	-24.33 – 3.62	0.146

N = 405, R^2 / R^2 adjusted = 0.276 / 0.240

Note: SAMS = Stendal Adherence to Medication Scale, BDI = Beck Depression Inventory, TuG = Time up and Go Test, MoCA = Montreal Cognitive Assessment, HCCQ = Healthcare Climate, Questionnaire, BFI = Big Five Inventory

Supplement to: The indirect effect of Nonadherence on Health-Related Quality of Life in Older Adults with Neurological Disorders: Implications for Clinical Endpoints and Interventions

Supplement Table 3. Linear Regression for MCS using SAMS and different covariates

**1**		**MCS**	
Predictors	*Estimates*	*CI*	*p*
(Intercept)	39.85	35.56 – 44.13	**<0.001**
SAMS	-0.23	-0.34 – -0.12	**<0.001**
HCCQ	1.91	1.19 – 2.63	**<0.001**
Observations	652		
R^2 / R^2 adjusted	0.075 / 0.072		

**2**		**MCS**	
Predictors	*Estimates*	*CI*	*p*
(Intercept)	41.56	34.37 – 48.76	**<0.001**
SAMS	-0.26	-0.37 – -0.15	**<0.001**
MoCA	0.38	0.08 – 0.68	**0.013**
Observations	689		
R^2 / R^2 adjusted	0.044 / 0.041		

**3**		**MCS**	
Predictors	*Estimates*	*CI*	*p*
(Intercept)	51.07	48.42 – 53.71	**<0.001**
SAMS	-0.28	-0.41 – -0.15	**<0.001**
TuG	-0.05	-0.28 – 0.17	0.636
Observations	449		
R^2 / R^2 adjusted	0.41 0.037		

**4**	**MCS**		
Predictors	*Estimates*	*CI*	*p*
(Intercept)	50.67	49.00 – 52.34	**<0.001**
SAMS	-0.33	-0.46 – -0.19	**<0.001**
Number of drugs	0.05	-0.18 – 0.28	0.678
Observations	639		
R^2 / R^2 adjusted	0.035 / 0.032		

Note: MCS = Mental Component Scale of SF-36, SAMS = Stendal Adherence to Medication Score, HCCQ = Healthcare Climate Questionnaire, MoCA = Montreal Cognitive Assessment, TuG = Timed Up and Go

Supplement to: The indirect effect of Nonadherence on Health-Related Quality of Life in Older Adults with Neurological Disorders: Implications for Clinical Endpoints and Interventions

Supplement Table 4. Linear Regression for MCS using Forgetting and different covariates

**1**		**MCS**	
Predictors	*Estimates*	*CI*	*p*
(Intercept)	57.80	56.69 – 58.91	**<0.001**
forgetting	-0.08	-0.37 – 0.21	0.608
BDI	-0.91	-1.00 – -0.82	**<0.001**
Observations = 689, R^2 adjusted= 0.380			

**2**		**MCS**	
Predictors	*Estimates*	*CI*	*p*
(Intercept)	57.80	56.69 – 58.91	**<0.001**
forgetting	-0.08	-0.37 – 0.21	0.608
BDI	-0.91	-1.00 – -0.82	**<0.001**
Observations = 689, R^2 adjusted = 0.380			

**3**		**MCS**	
Predictors	*Estimates*	*CI*	*p*
(Intercept)	38.58	31.49 – 45.66	**<0.001**
forgetting	-0.66	-1.02 – -0.31	**<0.001**
MoCA	0.49	0.19 – 0.79	**0.001**
Observations = 689, R^2 adjusted = 0.029			

**4**		**MCS**	
Predictors	*Estimates*	*CI*	*p*
(Intercept)	50.67	47.99 – 53.36	<0.001
forgetting	-0.64	-1.09 – -0.20	0.004
TuG	-0.08	-0.30 – 0.15	0.510
Observations = 449, R^2 adjusted = 0.015			

**5**		**MCS**	
Predictors	*Estimates*	*CI*	*p*
(Intercept)	50.15	48.50 – 51.81	<0.001
forgetting	-0.75	-1.15 – -0.34	<0.001
drugs	0.03	-0.20 – 0.26	0.808
Observations = 639, R^2 adjusted = 0.017			

Note: MCS = Mental Component Scale of SF-36, Forgetting = Stendal Adherence to Medication Score – Forgetting Subscale, HCCQ = Healthcare Climate Questionnaire, MoCA = Montreal Cognitive Assessment, TuG = Timed Up and Go

Supplement Table 5. Linear Regression for MCS using Missing Knowledge and different covariates

**1**	**MCS**		
Predictors	*Estimates*	*CI*	*p*
(Intercept)	57.83	56.75 – 58.92	**<0.001**
knowledge	-0.10	-0.30 – 0.11	0.368
BDI	-0.91	-1.00 – -0.82	**<0.001**
Observations = 689, adjusted = 0.381			

**2**	**MCS**		
Predictors	*Estimates*	*CI*	*p*
(Intercept)	37.94	33.77 – 42.10	**<0.001**
knowledge	-0.34	-0.60 – -0.08	**0.009**
HCCQ	2.11	1.39 – 2.83	**<0.001**
Observations = 652, R^2 adjusted = 0.057			

**3**	**MCS**		
Predictors	*Estimates*	*CI*	*p*
(Intercept)	40.83	33.20 – 48.46	**<0.001**
knowledge	-0.28	-0.56 – -0.01	**0.041**
MoCA	0.37	0.05 – 0.68	**0.023**
Observations = 689, R^2 adjusted = 0.016			

**4**	**MCS**		
Predictors	*Estimates*	*CI*	*p*
(Intercept)	50.03	47.41 – 52.64	**<0.001**
knowledge	-0.48	-0.81 – -0.14	**0.005**
TuG	-0.05	-0.27 – 0.18	0.687
Observations = 449, R^2 adjusted = 0.014			

**5**	**MCS**		
Predictors	*Estimates*	*CI*	*p*
(Intercept)	49.29	47.71 – 50.87	**<0.001**
knowledge	-0.34	-0.66 – -0.02	**0.040**
drugs	0.06	-0.18 – 0.30	0.631
Observations = 639, R^2 adjusted = 0.004			

Note: MCS = Mental Component Scale of SF-36, Knowledge = Stendal Adherence to Medication Score – Missing Knowledge Subscale , HCCQ = Healthcare Climate Questionnaire, MoCA = Montreal Cognitive Assessment, TuG = Timed Up and Go

Supplement to: The indirect effect of Nonadherence on Health-Related Quality of Life in Older Adults with Neurological Disorders: Implications for Clinical Endpoints and Interventions

Supplement Table 6. Linear Regression for MCS using Modification and different covariates

**1**	**MCS**		
Predictors	*Estimates*	*CI*	*p*
(Intercept)	57.80	56.74 – 58.86	**<0.001**
modification	-0.18	-0.39 – 0.04	0.103
BDI	-0.90	-0.99 – -0.81	**<0.001**

Observations = 689, R^2 adjusted = 0.383

**2**	**MCS**		
Predictors	*Estimates*	*CI*	*p*
(Intercept)	38.85	34.68 – 43.01	**<0.001**
modification	-0.55	-0.81 – -0.29	**<0.001**
HCCQ	1.96	1.24 – 2.68	**<0.001**

Observations = 652, R^2 adjusted = 0.073

**3**	**MCS**		
Predictors	*Estimates*	*CI*	*p*
(Intercept)	38.69	31.65 – 45.74	**<0.001**
modification	-0.61	-0.87 – -0.35	**<0.001**
MoCA	0.47	0.17 – 0.77	**0.002**

Observations = 689, R^2 adjusted = 0.040

**4**	**MCS**		
Predictors	*Estimates*	*CI*	*p*
(Intercept)	50.35	47.74 – 52.95	**<0.001**
modification	-0.58	-0.88 – -0.29	**<0.001**
TuG	-0.07	-0.30 – 0.15	0.527

Observations = 449, R^2 adjusted = 0.030

**5**	**MCS**		
Predictors	*Estimates*	*CI*	*p*
(Intercept)	50.19	48.57 – 51.81	**<0.001**
modification	-0.67	-0.97 – -0.38	**<0.001**
drugs	-0.05	-0.28 – 0.18	0.678

Observations = 639, R^2 adjusted = 0.027

Note: MCS = Mental Component Scale of SF-36, Modification = Stendal Adherence to Medication Score – Modification Subscale, HCCQ = Healthcare Climate Questionnaire, MoCA = Montreal Cognitive Assessment, TuG = Timed Up and Go

frontiers
